# Advances and prospects in fungal disease resistance breeding of roses

**DOI:** 10.7717/peerj.21666

**Published:** 2026-08-27

**Authors:** Xudong Zheng, Shuyue Cheng, Xueduo Li, Ge Yan, Shuhuan Zhang, Xu Deng, Chun Ou, Changbing Huang

**Affiliations:** 1 School of Biology and Food Engineering, Fuyang Normal University, Funan Rural Revitalization Collaborative Technology Service Center, Fuyang, Anhui, China; 2 Characteristic Flower Engineering Research Center of Jiangsu Province, Suzhou, Jiangsu, China; 3 Suzhou Polytechnic Institute of Agriculture, Suzhou, Jiangsu, China; 4 South Subtropical Crops Research Institute, Chinese Academy of Tropical Agricultural Sciences, Zhanjiang, Guangdong, China

**Keywords:** Rose, Fungal diseases, Disease resistance breeding, Research progress

## Abstract

*Rosa* species, as one of the most economically and ornamentally valuable flower groups worldwide, not only occupy a core position in the horticultural industry but also possess long-term substantial value for breeding applications. However, their production systems rely heavily on disease management. The persistent threats of fungal diseases, including black spot, powdery mildew, and gray mold, have severely impaired the ornamental quality and commercial value of rose plants, constituting a major constraint on the stable development of the rose industry. Compared with conventional hybrid breeding, molecular breeding allows targeted modification of specific heritable traits and plays a pivotal role in the development of disease-resistant rose cultivars. In recent years, with the rapid advancement of genomics, transcriptomics, and functional genomics, a growing number of gene resources and regulatory mechanisms associated with disease resistance in *Rosa* species have been progressively elucidated. This review systematically summarizes the pathogen characteristics, disease symptoms, and damage patterns of major fungal diseases affecting *Rosa* species, with a particular focus on research progress in disease-resistance-related transcription factors (*e.g*., WRKY, MYB, and bZIP), resistance genes (*e.g*., Rdr, MLO, and CPR), and key gene families identified from *Rosa chinensis* and *Rosa hybrida* materials. In addition, by integrating research evidence from model plants and major crops, this review comparatively analyzes the functional evolution of different regulatory factors and their underlying disease resistance mechanisms, thereby improving the understanding of the molecular regulatory network governing disease resistance in *Rosa* species. Furthermore, this review outlines the current applications and future potential of emerging molecular breeding technologies, such as Clustered Regularly Interspaced Short Palindromic Repeats (CRISPR)/CRISPR-associated protein 9 (Cas9), in the functional dissection of resistance genes and precise genetic improvement of roses. By systematically synthesizing major research achievements in the disease resistance of *Rosa* species, this review summarizes the current progress and existing challenges in relevant research and proposes future research directions for resistance gene mining, molecular regulatory network elucidation, and molecular design breeding. This study aims to provide a theoretical basis for the improvement of disease resistance and the breeding of new highly resistant cultivars of *Rosa* species.

## Introduction

Roses (*Rosa* spp.) is an important group of ornamental flowering plants with substantial economic and social value in landscape greening, cut-flower production, ecological landscape construction, and fragrance processing. This genus includes approximately 150–200 wild species. It is widely distributed throughout temperate and subtropical regions of the Northern Hemisphere. Asia, and China in particular, is considered an important center of origin and diversification for *Rosa* species, with abundant germplasm resources and high genetic diversity (Jian et al., 2022; Yu et al., 2014). In general, roses are armed shrubs or climbing plants. They are characterized by diverse flower colors, a wide range of flower forms, intense fragrance, and strong environmental adaptability. Thus, roses have attracted sustained attention from breeders and consumers. After centuries of artificial domestication and hybrid improvement, *Rosa* species have formed a large cultivated population. *Rosa chinensis* (*R. chinensis*) has long been prized for its ornamental qualities in China. Rose breeding received a boost when Chinese roses cultivars were introduced to Europe in the 18th century. Notable are the cultivars ‘Old Blush’, ‘Crimson China’, ‘Hume’s Blush Tea-scented China’, and ‘Park’s Yellow Tea-scented China’, which display important agronomic traits, such as recurrent flowering, increased floral fragrance, and improved disease resistance. The above-mentioned traits were subsequently introgressed into European rose germplasm *via* extensive hybridization with local native species, fundamentally reshaping the genetic basis of modern roses (Jian et al., 2010; Nakamura et al., 2018). Thanks to long-term domestication, hybridization, and selection, more than 35,000 modern rose cultivars have been developed and registered globally. These cultivars establish roses as one of the most widely cultivated ornamental crops and one of the most economically valuable floricultural species worldwide.

However, with the continuous expansion of the rose industry, various fungal diseases have occurred frequently in cultivation systems, posing a persistent threat to production stability. The primary fungal diseases affecting roses include black spot, powdery mildew, gray mold, and rust (Moral et al., 2018; Kumar, 2018). Fungal diseases in roses cause defoliation, which reduces plant photosynthetic area. Accordingly, plant vigor can be weakened, and ornamental value can be drastically diminished (Mekapogu et al., 2021). In Isparta, Turkey, the prevalence rate of rose rust reached 76.5% in 2011 and 81.9% in 2012, resulting in severe economic losses (Arıcı & Özkaya, 2014). In natural environments, the incidence of powdery mildew can reach up to 45.76% under high temperature and low relative humidity conditions (Kumar, Chandel & Kumari, 2016). Therefore, the development of disease-resistant germplasm resources is crucial for reducing maintenance costs and improving market competitiveness of roses.

The widespread prevalence and persistent damage of fungal diseases have severely compromised the ornamental value, cultivation efficiency, and sustainable development of roses. To preserve the high quality of roses and minimize disease-induced losses, chemical fungicides are widely adopted in practical production and remain the most prevalent disease control measure to date. However, the frequent fungicide applications required for rose disease management exert adverse impacts on ecological environments and increase production costs (Yasin et al., 2016). Both fungal infections and the corresponding fungicide applications substantially elevate the cultivation maintenance costs of *Rosa* species.

Researchers have been actively exploring alternative fungal disease control strategies to reduce dependence on chemical fungicides. Current studies mainly focus on the application of biological control agents and the breeding of disease-resistant rose varieties (McLaughlin et al., 2023; Windham et al., 2023). Biological control agents are generally defined as microorganisms that inhibit pathogenic microorganisms and serve as viable alternatives to synthetic chemical fungicides (Williams, Knox & Forsyth, 2025). Nevertheless, most biological control agents act on a single target site, rendering them vulnerable to the development of resistance in target pathogens (Jeschke et al., 2019). Similar to modern synthetic fungicides, most biological preparations function *via* a single molecular target, and their efficacy can therefore decline with the emergence of pathogen resistance. In this context, genetic improvement and disease-resistant cultivar breeding for roses constitute an effective strategy for long-term and sustainable disease management. Multiple research institutions and commercial breeding enterprises have successively launched systematic breeding programs focused on rose disease resistance. World-famous breeding companies, including Meilland, Kordes, and David Austin, have conducted successive rounds of hybridization to improve rose disease resistance and have released a large number of cultivars with excellent ornamental traits and strong disease resistance. A typical representative is the ‘Knock Out’ series developed by William Radler. This series exhibits prominent disease resistance and stable recurrent flowering performance under commercial conditions, verifying the successful transmission and improvement of resistance traits originally derived from Chinese roses.

At present, the breeding of *Rosa* species predominantly depends on conventional hybridization techniques. However, the long breeding cycle and high genetic uncertainty of traditional hybridization have driven the transformation of rose breeding strategies toward molecular breeding, which has been greatly facilitated by the progress of modern biotechnology. Molecular breeding overcomes multiple limitations of conventional breeding methods, such as the requirement for long-term phenotypic screening and restrictions on the utilization of genetic resources, enabling the precise cultivation of disease-resistant rose varieties (Wang & Zhang, 2022). Accordingly, this review summarizes the latest research advances in fungal disease resistance of *Rosa* species, with a particular focus on molecular mechanisms and breeding strategies. This study provides a theoretical basis for subsequent research on plant disease resistance and supports the development of precision breeding technologies for roses.

## Survey methodology

A systematic search strategy was implemented for a comprehensive and unbiased coverage of the literature. Relevant publications were retrieved from Web of Science, PubMed, Google Scholar, as well as Scopus. The search was performed within title, abstract, and keyword fields using the following Boolean string: (“Rosa” OR “rose” OR “Rosaceae”) AND (“fungal disease” OR “black spot” OR “powdery mildew” OR “Botrytis” OR “*Diplocarpon rosae*”) AND (“resistance” OR “tolerance” OR “defense response” OR “disease resistance genes” OR “R gene” OR “breeding” OR “marker-assisted selection”). The search was limited to articles published between 2002 and 2026. We initially retrieved 911 publications through the literature search. A total of 97 duplicate articles were excluded after duplicate records were removed based on titles and publication years. Consequently, 814 records were left for title and abstract screening. Only original experimental studies or breeding reports investigating fungal disease resistance in *Rosa* species were retained, provided such studies presented physiological, molecular, or genetic analyses of resistance, contained identification, mapping, or functional validation of resistance genes, and were written in English; review articles, conference abstracts, manuscripts without primary empirical data, studies exclusively focused on abiotic stress or non-fungal pathogens (unless fungal inoculation assays were additionally performed), and publications with unclear methodologies or genetic evidence were excluded. Full-text articles were acquired and thoroughly evaluated for all eligible studies. Subsequently, 32 review and review-style articles and 372 studies irrelevant to fungal disease resistance in rose were excluded. No qualifying books, book chapters, conference papers, or conference abstracts were identified throughout the screening procedure. For subsequent synthesis and analysis, we ultimately retained 410 original research articles. To reduce selection bias, two authors independently screened all titles and abstracts to evaluate study relevance. Disagreements regarding study eligibility were resolved *via* group discussion to reach mutual consensus. To further mitigate publication bias, the reference lists of all included articles were manually scanned to retrieve additional relevant studies omitted from the initial database search. This systematic, transparent approach ensured guaranteed comprehensive and reproducible documentation of the current research landscape concerning fungal disease resistance and resistance breeding in *Rosa* species.

## Major fungal diseases of roses and their associated pathogens

**Figure 1:**
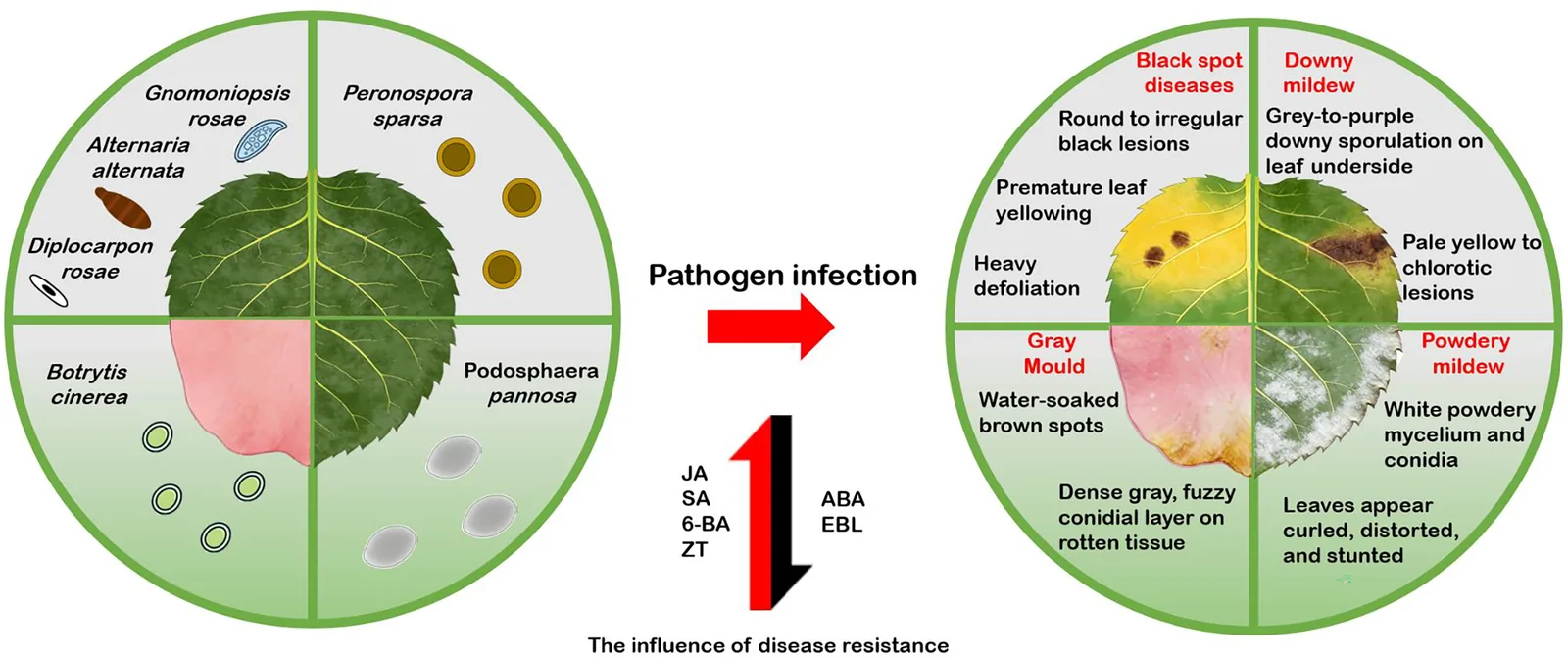
The influence of pathogen stress on roses. Black spot, powdery mildew, and downy mildew pathogens primarily infect rose leaves, whereas gray mold mainly affects rose petals. Exogenous application of plant hormones modulates plant disease resistance through various mechanisms. Specifically, jasmonic acid (JA), salicylic acid (SA), 6-benzylaminopurine (6-BA), and zeatin (ZT) enhance disease resistance in roses, while abscisic acid (ABA) and epibrassinolide (EBL) compromise it.

Pathogen infection is closely associated with the dynamic balance between autophagy and apoptosis in host cells. During the early stages of colonization, pathogens may suppress autophagy, an intrinsic cellular homeostasis and damage-repair mechanism, The suppression aims at maintaining host cell viability and establishing a favorable niche for the replication of pathogens. As host-pathogen interactions progress, pathogens can activate apoptotic pathways, thereby inducing programmed cell death and subsequent tissue necrosis (Veloso & van Kan, 2018). Table 1 lists the most commonly reported fungal pathogens infecting *R. chinensis*. The effects of pathogen stress on *R. chinensis* are depicted in Fig. 1. Table 1 further details the major pathogens, affected plant organs, and yield loss rates associated with the seven primary fungal diseases of roses (Bagsic, Linde & Debener, 2016; Kang et al., 2019; Neu et al., 2017; Suthaparan et al., 2010; Ullah et al., 2024; Wilson & Aime, 2014). For instance, black spot disease primarily infects leaves but causes the most severe yield loss (a maximum of 67%). As indicated by this data, premature and extensive defoliation severely impairs photosynthetic capacity. Accordingly, cut flower yield and overall flowering performance can be reduced. The distribution, severity, and detrimental impacts of these pathogens are strongly modulated by environmental conditions, particularly temperature and humidity. Once infection is initiated, diseases can spread rapidly across field populations, and even minor lesions on leaves or flowers may lead to substantial yield losses (Debener & Byrne, 2014).

**Table 1:** The main pathogenic fungi infecting roses and their impacts on crops.

Disease	Foliar pathogens	Principal sites of impact	Yield losses	Reference
Black spot disease	*Diplocarpon rosae*	Blades	67%	Neu et al. (2017)
Powdery mildew	*Erysiphaceae*	Foliage and flower buds	20%~25%	Suthaparan et al. (2010)
Gray mould	*Botrytis cinerea*	Floral buds and petals	Significantly affect	Ullah et al. (2024)
Anthracnose	*Dothideomycetes*	Foliage and branches	Significantly affect	Bagsic, Linde & Debener (2016)
Cercospora leaf spot	*Cercospora rosicola*	Mature leaves	Significantly affect	Kang et al. (2019)

### Gray mold

Gray mold is a devastating plant disease primarily caused by *Botrytis cinerea* (*B. cinerea*), a prevalent necrotrophic fungal pathogen that spreads mainly *via* airborne spores. Its disease incidence rises markedly under high-humidity conditions (Motlagh & Jafari, 2022; Muñoz, Faust & Schnabel, 2019). *B. cinerea* predominantly survives in the form of mycelia and conidia, and can also persist as sclerotia in residual plant debris. Owing to its versatile infection strategies, *B. cinerea* is capable of infecting more than 400 crop species globally (Williamson et al., 2007). As a typical necrotrophic pathogen, *B. cinerea* exhibits an initial 8–16 h biotrophic-like growth phase at the early stage of infection. During this stage, the pathogen sustains host cell viability by inhibiting host autophagic processes. Following this initial phase, the fungus differentiates specialized infection structures and secretes toxins and cell wall-degrading enzymes, which trigger host cell apoptosis. Host cell death further leads to cell wall degradation and tissue necrosis, releasing nutrients that support the continuous colonization and proliferation of the pathogen (Veloso & van Kan, 2018).

Disease incidence is highest in petals compared with stems, stamens, ovaries, sepals and leaves, all of which exhibit browning and necrosis following infection (Ullah et al., 2024). In buds, infection initially appears as small irregular spots on the receptacle that enlarge into gray-black lesions, subsequently spreading across the bud, inhibiting opening, and leading to browning, rot, and collapse. In leaves, pale-brown irregular lesions typically develop at leaf margins and apices, becoming sunken, darkening, and eventually resulting in tissue decay. Magenta-, brown-, or white-tinged spots In petals enlarge into water-soaked or necrotic lesions, followed by tissue shriveling and rot (Ha et al., 2022; Motlagh & Jafari, 2022). Cao et al. (2019) assessed disease resistance of *Rosa hybrida* (*R. hybrida*) cultivars through a detached petal-disc assay. *B. cinerea* isolates were grown on potato dextrose agar (PDA) for 10–14 days. Conidia were collected in half-strength potato dextrose broth (PDB) and adjusted to a final concentration of 1 × 10^5^ conidia mL^−1^. Fifteen-millimeter discs excised from the outermost petals were placed on 0.4% (w/v) agar and then inoculated with 2 µL of the conidial suspension. Differences in lesion development became evident at 60–72 h post-inoculation. This assay was subsequently adapted to assess the effects of phytohormones on petal disease resistance. For hormone treatments, cut flower stems were exposed for 24 h to either 50 µM jasmonic acid (JA) or 50–400 µM 1-aminocyclopropane-1-carboxylic acid (ACC); whole flowers were also incubated for 24 h under atmospheres containing ethylene (ET) (10 µL·L^−1^) or 1-MCP (2 µL·L^−1^). The authors demonstrated that the activation of ET signaling (*via* ACC or ET exposure) and JA treatment significantly enhanced the resistance of rose petals to *B. cinerea*. Notably, the inhibition of ET perception by 1-MCP reduced disease resistance, supporting the protective role of ET signaling in rose disease resistance. Liu et al. (2023) treated detached petals for 24 h with 100 µM 6-benzylaminopurine (6-BA), 100 µM zeatin (ZT), or 100 µM abscisic acid (ABA). They reported that 6-BA and ZT enhanced the disease resistance of *R. hybrida*, whereas ABA treatment promoted pathogen invasion.

### Black spot disease

Black spot disease is primarily caused by *Alternaria* spp. and other fungal pathogens, and it is a globally prevalent plant disease that markedly reduces crop yield as well as the ornamental and commercial value of horticultural species (Al-lami et al., 2023). The species composition of black spot pathogens varies across regions due to divergent geographical and climatic conditions. The major pathogens responsible for black spot disease in *R. chinensis* include *Marssonina rosae* (*M. rosae*), *Alternaria alternata* (*A. alternata*), and *Gnomoniopsis rosae* (*G. rosae*) (Li et al., 2023b; Neu et al., 2017). Among these pathogens, *G. rosae* was first identified as a causal agent of rose black spot disease in Yunnan Province, China (Li et al., 2023b).

*Alternaria* spp. are typical necrotrophic fungal pathogens that primarily infect *R. chinensis* leaves. These pathogens can spread to stems, fruits, and sepals under severe conditions (Yasin et al., 2016). In roses, pathogenic fungal infection exhibits the initial appearance of irregular purple-to-black necrotic lesions on leaves. As the disease progresses, chlorosis develops in the surrounding tissues, lesions enlarge and coalesce. Consequently, premature defoliation, branch necrosis, morphological abnormalities, and a marked decline in overall plant vigor can be triggered (Dong et al., 2017). Detached-leaf inoculation assays in roses can be carried out using a conidial suspension. As indicated by experimental results, inoculation with a suspension adjusted to 1 × 10⁵ conidia mL^−1^ could result in statistically significant lesion development after 8 days. Alternatively, inoculation can be achieved by placing mycelial plugs directly onto the leaf surface (Cheng et al., 2022; Yang et al., 2024). Li et al. (2023b) inoculated 5-mm-diameter mycelial plugs onto wounded sites of detached leaves. The plugs were covered with alcohol-sterilized cotton balls, and the leaves were misted daily to maintain high humidity. Pronounced disease symptoms became evident 7 days post-inoculation (Li et al., 2023b). Song et al. (2024) applied a concentration series of 24-epibrassinolide (EBL; 0.1, 0.5, 1.5, 3, and 6 mg·L^−1^) to rose leaves and observed an unexpected increase in plant susceptibility to black-spot disease. It is noteworthy that the roles of other phytohormones in modulating rose black spot disease remain unclear.

### Powdery mildew

*Podosphaera pannosa* (*P. pannosa*) is a biotrophic fungal pathogen belonging to the phylum Ascomycota and the order Erysiphales. It serves as the causal agent of powdery mildew in *R. chinensis* (Suthaparan et al., 2012). Powdery mildew primarily colonizes the surfaces of various *R. chinensis* organs (*e.g*., young and mature leaves, stems, and flower buds). Its spore production and release occur independently of free liquid water, enabling efficient dissemination throughout its life cycle even under arid or low-humidity conditions (Suthaparan et al., 2010). Conidia land on the host’s epidermis during the early stage of infection. Under optimal temperatures, conidia germinate to produce germ tubes that penetrate the cuticle and form haustoria for nutrient uptake. Intercellular hyphae subsequently proliferate and produce conidiophores, whose apices generate chains of new conidia. The characteristic powdery coating results from prolific conidial production and facilitates subsequent infection cycles. Infected tissues undergo desiccation, wilting, and necrosis as nutrients are siphoned off, severely reducing the ornamental value of *R. chinensis*.

These abundant conidia form distinct white powdery lesions on leaf surfaces, particularly on the abaxial side in the early infection stage, marking the onset of the symptomatic phase (Karami et al., 2024). Studies have shown that under conditions of 20 °C and 100% relative humidity, *P. pannosa* conidia reach their maximum germination rate within 2–4 h, whereas germination is markedly inhibited when the temperature approaches 30 °C (Domínguez-Serrano et al., 2016). Consequently, in regions with short, rapidly warming springs, such as Northeast China and inland areas of New England, the persistent 15–25 °C high-humidity window suitable for infection is rarely sustained, resulting in a relatively low natural epidemic risk of rose powdery mildew (Belanger et al., 2002). *In vitro* resistance assays for rose powdery mildew commonly adopt artificial inoculation *via* uniform spraying of spore suspensions onto leaf surfaces to simulate natural infection and ensure full exposure to pathogenic spores. Maintaining high relative humidity during this procedure is critical, as it substantially promotes spore germination and subsequent fungal invasion (Yan et al., 2006). Liu et al. (2022) treated rose leaves with ACC (an ET precursor), gaseous ET, or 1-MCP (anET receptor inhibitor), followed by inoculation with *P. pannosa*. Their results demonstrated that 1-MCP treatment enhanced plant resistance to powdery mildew, whereas ACC application increased host susceptibility, indicating that ET signaling plays a critical regulatory role in mediating plant susceptibility to this disease. In an independent study, Yang et al. (2022) performed exogenous salicylic acid (SA) treatment on *R. chinensis* and compared transcriptomic and metabolomic responses following *P. pannosa* infection. They found that both SA treatment and pathogen infection activated overlapping defense-related pathways, including MAPK signaling, R-gene expression, and secondary metabolite biosynthesis, suggesting that SA acts as an inducer that mimics or enhances plant immune responses.

## The disease resistance mechanism of *Rosa* species

### The immune response of plants

**Figure 2:**
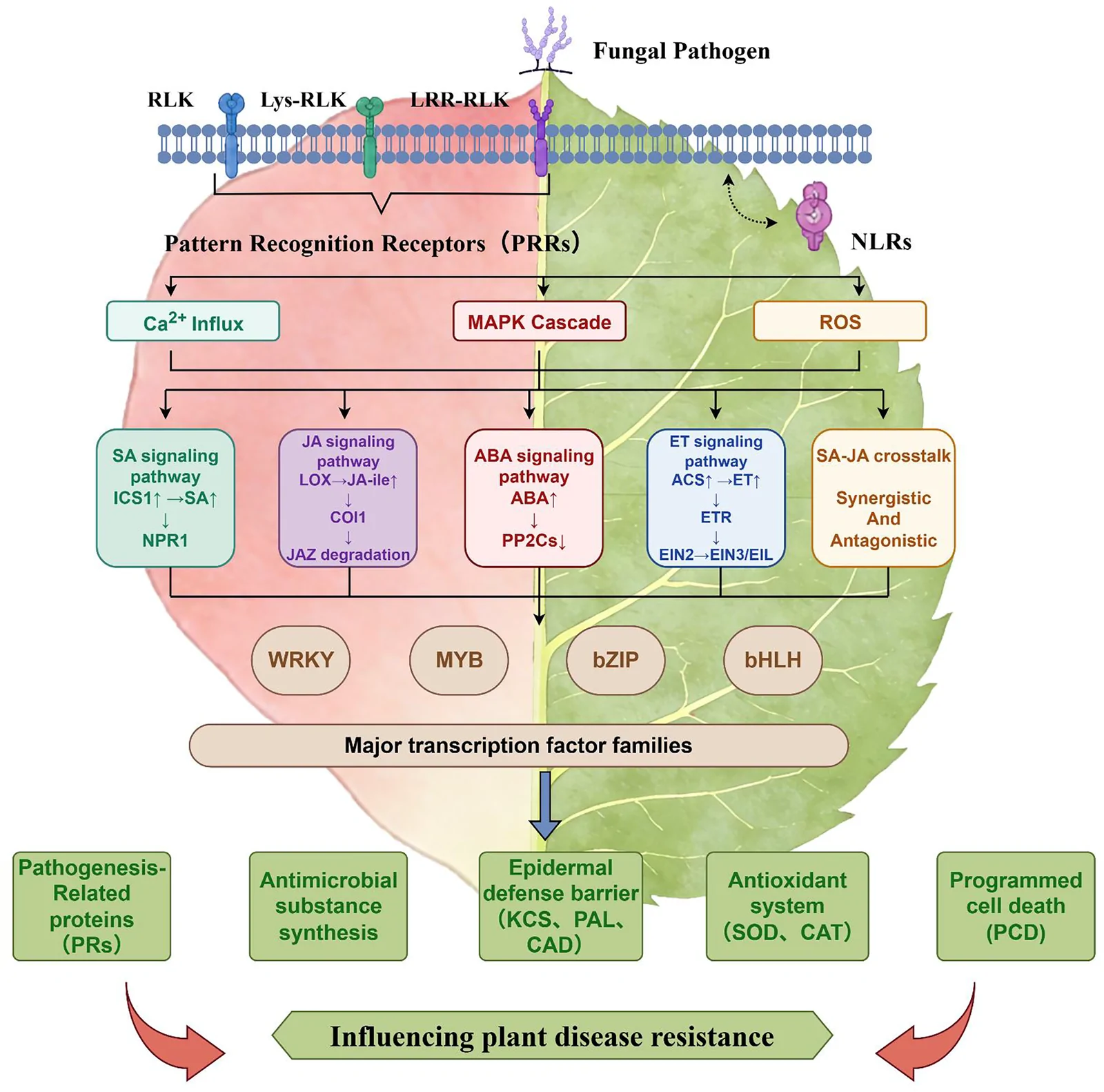
Proposed immune regulatory framework of rose responses to fungal pathogen infection. Pathogen recognition by PRRs and NLRs initiates early immune signaling events, including Ca^2+^ influx, ROS production, and MAPK activation. These signals modulate SA, JA, ET, and ABA pathways and their interactions, which subsequently regulate key transcription factor families such as WRKY, MYB, bZIP, and bHLH. The activated transcription factors orchestrate downstream defense-related genes involved in PR protein accumulation, antimicrobial metabolite biosynthesis, cell wall reinforcement, antioxidant responses, and programmed cell death, thereby contributing to enhanced disease resistance in roses.

To provide an integrated view of roses immune regulation, the sequential signaling cascade from pathogen perception to hormone-mediated transcriptional reprogramming and downstream defense responses is summarized in Fig. 2. The waxy cuticle covering the outer surface of cell walls represents the first line of physical defense in plants. When this physical barrier is breached, plants rapidly perceive conserved pathogen-associated molecular patterns (PAMPs) through cell surface-localized pattern recognition receptors (PRRs). Thus, the primary immune response, known as pattern-triggered immunity (PTI), can be activated. However, certain pathogens overcome this defense layer by delivering effector proteins into host cells to suppress PTI (Huang et al., 2023; Liu et al., 2018). In response, plants activate effector-triggered immunity (Cheng et al., 2025a), a secondary defense mechanism mediated by disease-resistance genes that encode intracellular nucleotide-binding leucine-rich repeat receptors (NLRs). These receptors directly or indirectly recognize pathogen effectors, thereby enhancing plant resistance to pathogen invasion (Huang et al., 2023).

PTI serves as the primary layer of host defense against pathogen invasion and initiates a cascade of cellular and physiological responses. The above-mentioned responses encompass Ca^2+^ influx, extracellular alkalinization, membrane depolarization, ion efflux, the production of nitric oxide (NO), reactive oxygen species (ROS), and phosphatidic acid (PA), the activation of an evolutionarily conserved mitogen-activated protein kinase (MAPK) cascade, ET biosynthesis, callose deposition, and extensive transcriptional reprogr ET amming. These responses can collectively restrict pathogen proliferation (Wang et al., 2020; Wu, Shan & He, 2014). PTI and Effector-Triggered Immunity (ETI) are tightly interconnected. This close connection is achieved as PRRs and NLRs mediate overlapping downstream immune responses (*e.g*., Ca^2+^ influx and ROS bursts). Ca^2+^ influx activates plasma membrane-localized Nicotinamide Adenine Dinucleotide Phosphate (NADPH) oxidases (respiratory burst oxidase homologs, RBOHs), leading to ROS production. In turn, ROS modulate membrane potential and ion channels, further promoting Ca^2+^ influx and establishing a positive feedback loop. Collectively, Ca^2+^ and ROS signaling activate the MAPK cascade (MAPKKK→MAPKK→MAPK), which transduces defense signals *via* the phosphorylation of intracellular targets. Accordingly, cell wall reinforcement and the induction of defense-related gene expression can be achieved (Ding et al., 2022; Negi et al., 2023). Transcriptomic analyses of *R. chinensis* have revealed that fungal infection triggers the MAPK cascade, causing significant upregulation of key cascade components such as *MPK3* and *MPK6* (Gao et al., 2021; Liu & He, 2017). Nevertheless, PTI and ETI differ in the timing, amplitude, and duration of their responses, and these discrepancies are critical for determining distinct physiological outcomes. For instance, ETI frequently induces rapid localized programmed cell death at infection sites, a phenomenon termed the hypersensitive response (HR). Overall, ETI elicits a stronger and more specific immune response than PTI (Huang et al., 2023).

### Roles of transcription factors (TFs) in disease resistance of *Rosa* species

**Figure 3:**
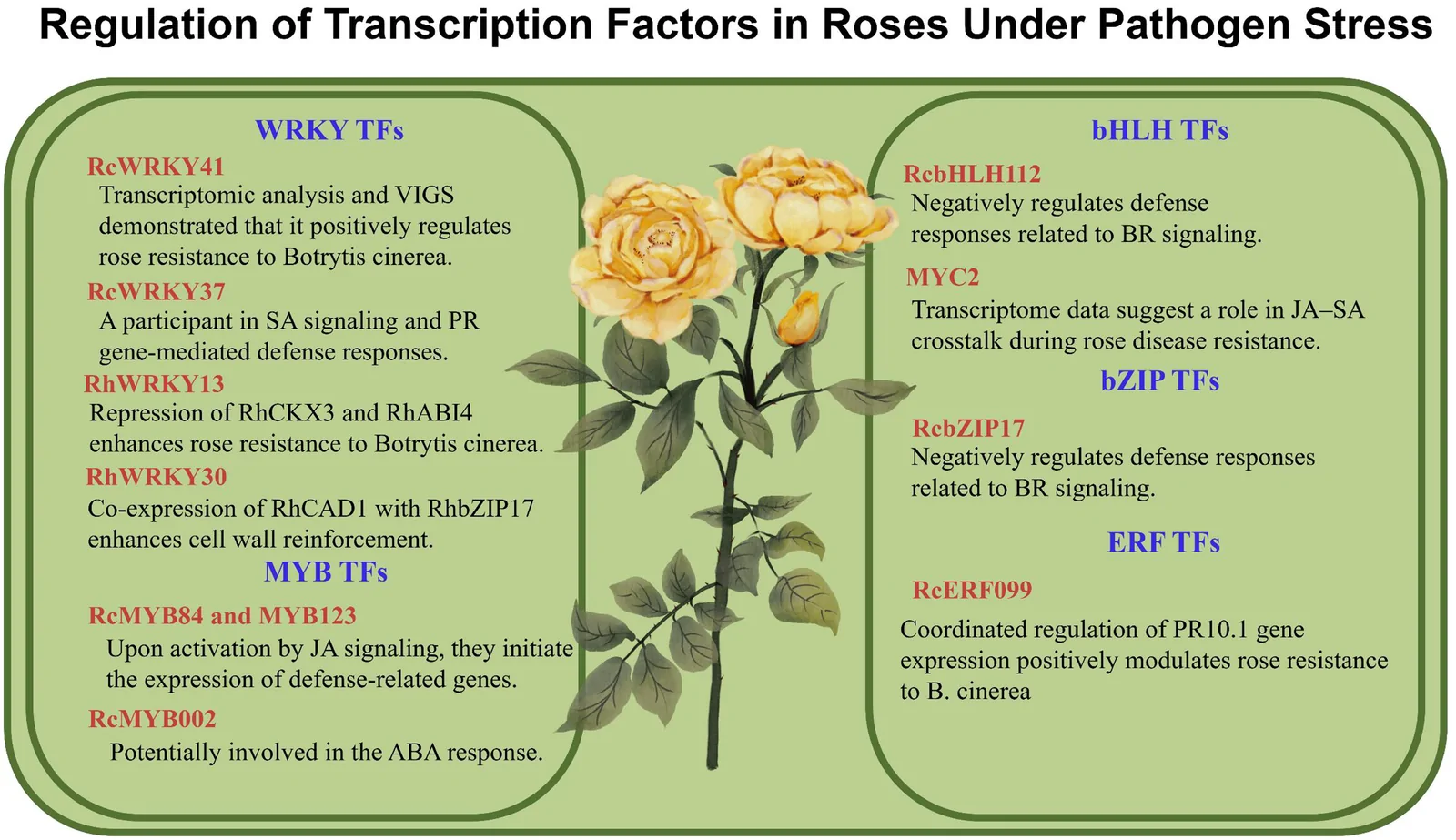
Major transcription factor families associated with disease resistance and their regulatory functions. Transcription factor families, including WRKY, MYB, bHLH, bZIP, and ERF, participate in the regulation of rose immunity through multiple signaling pathways. These factors modulate defense-related gene expression, hormone signaling (SA, JA, ABA, and BR), PR protein accumulation, and cell wall reinforcement, thereby contributing to resistance against pathogens such as *Botrytis cinerea*. Positive and negative regulatory roles of representative transcription factors identified in rose are summarized.

Transcription factors (TFs) act as core regulatory nodes that connect the downstream responses of PTI and ETI signaling pathways. The activation of PTI or ETI triggers a series of signaling cascades, which ultimately leads to the activation or repression of specific TFs. These TFs subsequently translocate into the nucleus and bind to the promoter regions of target genes to initiate or suppress gene transcription. Transcriptomic analysis of rose petals infected with *B. cinerea* has identified 188 upregulated TF-encoding genes, which are significantly enriched in the ERF, WRKY, bHLH, MYB, and NAC families (Liu et al., 2018). Transcriptomic profiling of rose leaves infected with powdery mildew has uncovered 1,181 differentially expressed genes (DEGs). Among these DEGs, TFs belonging to the ERF, MYB, bHLH, and WRKY families are detected, most of which are downregulated during *P. sparsa Berk*. infection (Liu et al., 2022). Accordingly, this section comprehensively reviews TFs associated with disease resistance in roses and their underlying regulatory mechanisms. Figure 3 illustrates the regulatory mechanisms of TFs in roses under pathogenic fungal stress.

The WRKY transcription factor family, which functions as either transcriptional activators or repressors, serves as a crucial component of plant regulatory networks. To date, the identified members of the WRKY family in roses have expanded from 56 to 68, and substantial studies have focused on their functions in biotic and abiotic stress responses (Liu et al., 2019; Yan et al., 2024). Multiple transcriptomic studies have demonstrated that numerous WRKY family members are extensively involved in the modulation of disease resistance in roses following pathogen infection, with distinct and variable expression patterns across different infection contexts (Domes & Debener, 2024; Song et al., 2024). Most currently reported WRKY transcription factors (WRKY TFs) in roses are induced by pathogen infection and mediate plant disease resistance *via* hormone-dependent signaling pathways and/or the reinforcement of cell wall-related defense responses. Nevertheless, individual WRKY family members exhibit evident functional diversification in their regulatory modes and downstream signaling pathways. Zheng et al. (2024b) reported that RcWRKY40 enhanced resistance to black spot disease. This enhancement was achieved by facilitating the SA signaling pathway and suppressing the JA signaling pathway. Thus, the antagonistic effect of JA on SA signaling can be alleviated. Moreover, RcWRKY40 affected plant susceptibility or resistance by altering the accumulation level of auxin (IAA). Notably, this regulatory mechanism is not unique to rose. A similar mode of action was identified in early research on *Arabidopsis thaliana* (*A. thaliana*). In *A. thaliana*, WRKY70 has become a critical cross-regulatory hub between the SA and JA signaling pathways in plant defense responses. The cross-regulatory role of WRKY70 can be achieved *via* its SA-inducible and JA-repressible expression pattern, as well as its dual regulatory function of activating SA signaling and suppressing JA signaling. This is highly similar to the mechanism of WRKY40 in rose, further suggesting that WRKY transcription factor-mediated SA-JA crosstalk may be evolutionarily conserved across the plant kingdom (Li, Brader & Palva, 2004). As indicated by early studies, AtWRKY33 can facilitate the expression of defense-related genes (*e.g*., *PDF1.2*) by mediating JA-dependent defense pathways, thereby enhancing plant resistance to *B. cinerea* and *Alternaria brassicicola* (*A. brassicicola*); moreover, its overexpression suppresses SA-dependent *PR-1* expression and increases susceptibility to *Pseudomonas syringae* (*P. syringae*) (Zheng et al., 2006). Mechanistically, AtWRKY33 functions downstream of the MAPK signaling cascade. Upon pathogen recognition, MPK3, MPK4, and MPK6 phosphorylate AtWRKY33, leading to its activation and the subsequent induction of defense-related genes. Activated AtWRKY33 directly regulates genes involved in phytoalexin biosynthesis. In particular, these genes cover *CYP71B15* (*PAD3*), a key gene in camalexin biosynthesis, which can facilitate the accumulation of antimicrobial compounds and restrict pathogen colonization (Mao et al., 2011). Subsequent studies showed that AtWRKY33 also enhances resistance to *B.cinerea* by directly repressing the expression of the key ABA biosynthetic genes *NCED3* and *NCED5*, thereby limiting ABA accumulation (Liu et al., 2015). In the phylogenetic analysis constructed by Liu et al. (2019) during the genome-wide screening of the rose WRKY family, RcWRKY41 was found to be closely related to AtWRKY33. After *B. cinerea* infection, expression analysis and virus-induced gene silencing assays indicated that petals with silenced RcWRKY41 exhibited more severe disease symptoms. As revealed by this result, RcWRKY41 can play a critical positive regulatory role in rose resistance to gray mold. The regulatory mechanism of RcWRKY41 may be similar to that of AtWRKY33. However, no relevant experimental evidence has yet been obtained. RhWRKY13 enhances endogenous cytokinin (CK) levels in *R. hybrida* by directly binding to and repressing the promoter activity of *RhCKX3*, thereby reducing CK degradation. Concurrently, it suppresses the expression of *RhABI4*, a key component of the ABA signaling pathway. Thus, ABA signal transduction can be attenuated. Through these coordinated regulatory mechanisms, *RhWRKY13* positively regulates *R. hybrida* resistance to *B. cinerea* (Liu et al., 2023). Remarkably, cooperative regulation between WRKY TFS and other transcription factors is common. To be specific, OsWRKY62 and OsWRKY76 TFs form both homomeric and heteromeric complexes in the nucleus, and achieve negative regulation of *Oryza sativa* (*O. sativa*) responses to *Magnaporthe oryzae* (*M. oryzae*) and *Xanthomonas oryzae* (*X. oryzae*) stresses through physical interaction with the *O. sativa* importins OsIMα1a/1b *via* a novel nuclear localization signal in the WRKY domain (Liu et al., 2016; Xu et al., 2022). OsMYB30 promotes lignin and SA biosynthesis by binding to AC-like elements in the promoters of *OsPAL6* and *OsPAL8*, whereas OsWRKY36 negatively regulates the PAL pathway. They constitute a finely tuned disease-resistance regulatory network (Liu et al., 2025). Similarly, Hu et al. (2021) observed the above phenomenon in *R. hybrida*: RhWRKY30 and RhbZIP17 were co-induced in response to *B. cinerea*. As a result, the transcriptional activity of the lignin biosynthetic gene *RhCAD1* was significantly enhanced. Accordingly, the structural barrier was strengthened, and resistance to pathogen invasion was enhanced.

Roses MYB transcription factors have been considered essential regulatory nodes linking hormone signaling to defense responses over the past few years. In *R. chinensis*, a total of 100 MYB family members have been identified, and the promoter regions of these genes are enriched in ABA-responsive elements; notably, ABA treatment can strongly induce the expression of RcMYB002, suggesting that it may participate in ABA-mediated stress responses and defense regulation (Zhu et al., 2024). Ren et al. (2020) demonstrated through VIGS that RcMYB84 and RcMYB123 positively regulate rose resistance against gray mold disease, and that these transcription factors play key roles in JA-induced immune responses. In the absence of JA signaling activation, JAZ1 can directly interact with RcMYB84/RcMYB123 to suppress the expression of downstream defense genes. Once JA signaling is activated, the degradation of JAZ1 releases the MYB regulatory complex, thereby initiating defense programs and enhancing plant disease resistance. In addition, Zhang et al. (2024b) showed that RcMYB8 not only responds to salt and drought stress, but also directly binds to the promoters of RcPR5/1 and RcP5CS1, thereby promoting proline accumulation, enhancing ROS scavenging capacity, and improving stress tolerance, indicating that rose MYBs may exert dual functions in both abiotic stress adaptation and immune regulation.

Such regulatory mechanisms are also widespread in other plant species. In *Arabidopsis thaliana*, MYB108 is upstream regulated by JA signaling and participates in the integration of defense responses under *B. cinerea* infection, as well as drought, high-salt, and oxidative stress conditions. MYB96 is associated with SA biosynthesis through the ABA pathway. Thus, drought tolerance and disease resistance can be coordinated (Seo & Park, 2010; Seo et al., 2009). Moreover, the promotion of lignin biosynthesis enables AtMYB20/42/43 to strengthen the cell wall barrier (Geng et al., 2020). Overall, rose MYB transcription factors likely integrate ABA and JA signaling pathways and coordinate the expression of PR genes, ROS homeostasis, and secondary metabolic pathways, thereby improving disease resistance under pathogen stress. This regulatory pattern is highly consistent with the general function of MYBs as core hubs connecting hormone signaling and defense metabolism in other plant species, although the regulatory network of this class of transcription factors in rose remains insufficiently characterized and requires further investigation.

Basic helix-loop-helix (bHLH) TFs constitute another important class of transcriptional regulators involved in plant immunity. As revealed by previous genome-wide identification research, the bHLH transcription factor family comprises 142 members in *Rosa persica* (*R. persica*) and 100 members in *R. chinensis*. Moreover, upon *B. cinerea* infection, several Rc-bHLH genes exhibit significant differential expression between resistant and susceptible rose cultivars at varying time points (Ullah et al., 2022; Zhuang et al., 2024). Through virus-induced gene silencing (VIGS), Ding et al. (2023) further validated the function of RcbHLH112. Silencing of *RcbHLH112* significantly enhanced rose resistance to *B. cinerea*, indicating that this gene acts as a negative regulator of defense in this pathosystem. MYC2 belongs to the MYC subgroup of the bHLH transcription factor family and functions as a central transcriptional activator in the JA signaling pathway (Wu & Ye, 2020). Gong et al. (2024) showed that RhMYC2, by directly regulating the CKbiosynthetic gene (*RhLOG3*) and the hydrolase gene (*RhCKX6*), coordinates JA and CK signaling to control petal size. This finding indicates that *R. hybrida* bHLH members can interact with multiple hormone-signaling networks. At present, studies directly addressing the mechanisms of *R. hybrida* bHLH factors under abiotic stress remain limited; however, existing evidence suggests that bHLH TFs mainly confer disease resistance by regulating JA signal transduction, ROS homeostasis, and defense-related secondary metabolism. For example, MYC2-like bHLH TFs, as core transcriptional regulators of the JA signaling pathway, can activate defense genes, phytoalexin biosynthesis, and the phenylpropanoid metabolic pathway (Ogawa et al., 2017). In contrast to MYC-type bHLH TFs, which act as positive regulators, tomato bHLH130 was identified as a negative regulator of late blight resistance. Silencing of bHLH130 enhances ROS accumulation and PR gene expression, and may also regulate plant immunity through the JA signaling and phenylpropanoid metabolism pathways, thereby increasing tomato resistance to *Phytophthora infestans* (Wang et al., 2025a). More recent studies have revealed that rice bHLH25 can directly sense H₂O₂ and coordinate lignin and phytoalexin biosynthesis, thereby revealing a new mechanism by which bHLH transcription factors participate in plant immunity as redox sensors (Liao et al., 2025). Collectively, these studies provide an important theoretical basis for elucidating the disease-resistance functions of bHLH transcription factors and their regulatory networks in roses.

Basic leucine zipper (bZIP) TFs constitute one of the largest transcription factor families in plant genomes. A genome-wide analysis of *R. chinensis* identified a total of 64 RcbZIP transcription factor family members, and moreover, several of these members may play key roles in the salt-stress response of *R. chinensis* (Cheng et al., 2024). Compared with WRKY and MYB TFs, research on bZIP transcription factors in rose disease resistance has progressed relatively late; apart from the above-mentioned synergistic interaction between RhbZIP17 and RhWRKY30, there are almost no such studies in *Rosa* species. Nevertheless, it is undeniable that bZIP transcription factors play an important role in plant defense against biotic stresses. Gao et al. (2021) demonstrated that RcTGA1, a bZIP family transcription factor, positively regulates rose resistance to *B. cinerea* through glucosinolate biosynthesis pathway modulation, as evidenced by VIGS silencing experiments (Gao et al., 2021). The TGA/OBF subgroup of bZIP transcription factors functions as key regulators of SA-dependent plant immunity, interacting physically with NPR1 to activate defense gene expression (Um, Lee & Seo, 2026). SA accumulation induces redox-mediated oligomer-to-monomer conversion of NPR1, triggering its nuclear translocation. In the nucleus, NPR1 forms complexes with TGA factors that bind cis-elements in promoters of *PR* genes, driving upregulation of *PR1*, *PR2*, and *PR5* (Zhang et al., 2025a). This NPR1-TGA regulatory module is of vital importance in SA-induced systemic acquired resistance (SAR) against biotic stresses (Guan et al., 2023). The defense functions of many bZIP family members, such as GmbZIP19, AtTGA, and OsTGA2, have been extensively studied, particularly in *A. thaliana* (He et al., 2020; Kesarwani, Yoo & Dong, 2007; Moon et al., 2018).

Current evidence suggests that rose disease resistance is controlled by a multilayered transcriptional regulatory network. WRKY TFs mainly regulate defense signaling pathways and pathogen-responsive genes; MYB transcription factors coordinate secondary metabolism; bHLH TFs may modulate hormone signaling and ROS homeostasis; and bZIP TFs participate in structural defense through lignin biosynthesis and cell wall reinforcement. Nevertheless, research on disease-resistance TFs in roses is still in its early stages. Most functional studies have focused on resistance to *B. cinerea*, whereas the transcriptional regulation of resistance to black spot, powdery mildew, and other economically important pathogens remains poorly understood. In addition, the downstream target genes and protein interaction networks of most rose TFs have yet to be elucidated.

### Expression of disease-resistant related gene family

**Figure 4:**
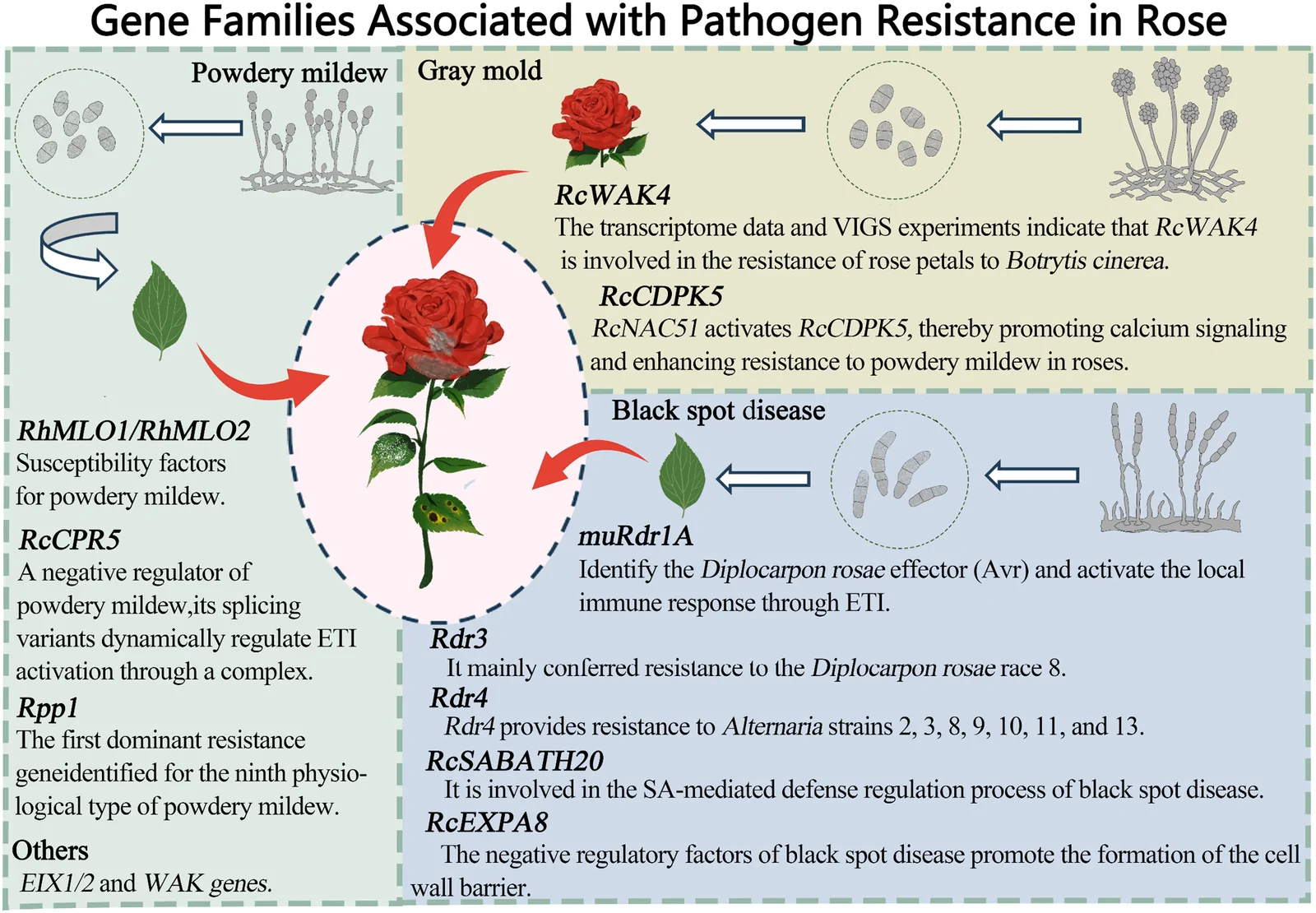
Gene families associated with fungal disease resistance in roses and their current research status. The major gene families implicated in resistance to black spot, powdery mildew, and gray mold in roses. Some gene families have been functionally characterized through genetic transformation, gene silencing, and other molecular approaches, providing insights into their roles in immune regulation. In contrast, other gene families have been identified mainly through transcriptomic and differential expression analyses and remain to be functionally validated. Nevertheless, their expression patterns suggest potential roles in disease responses and defense regulation, making them promising candidates for future studies on fungal disease resistance in roses.

Increasing evidence indicates that multiple gene families contribute to fungal disease resistance in roses. Nevertheless, the current understanding of their biological functions remains uneven, with only a subset having been experimentally validated, while many others are supported primarily by transcriptomic and differential expression analyses. Figure 4 provides a comprehensive summary of the major gene families associated with resistance to black spot, powdery mildew, and gray mold in roses. Representative gene families and their regulatory roles in disease resistance are discussed in the following sections.

Among the numerous rose genes associated with resistance to black spot disease, members of the *Rdr* (Resistance to *Diplocarpon rosae*) gene family, particularly *Rdr1*, play a central role. The *Rdr1* locus, located in the telomeric region of rose chromosome 1, comprises a young cluster of highly similar TIR-NBS-LRR (Toll/interleukin-1 receptor-nucleotide-binding site-leucine-rich repeat, TNL) candidate genes rather than a single gene (Diaz-Pendon et al., 2007). This locus encodes canonical intracellular NLR (nucleotide-binding leucine-rich repeat) immune receptor proteins. Its mode of action conforms to the classical gene-for-gene model, whereby *Rdr1* specifically recognizes a corresponding effector (avirulence factor) secreted by *Diplocarpon rosae* (*D. rosae*), thereby triggering a robust defense response (Arias, 2021). In general, this recognition induces an HR at the infection site, characterized by localized programmed cell death. Accordingly, haustorium formation can be prevented, and pathogen spread can be restricted (Terefe-Ayana et al., 2011). Consequently, *Rdr1* confers broad-spectrum resistance against multiple pathogen isolates; however, this resistance can be overcome by emerging races carrying corresponding virulence alleles (Wang et al., 2010). In addition to *Rdr1*, other loci, including *Rdr2*, *Rdr3*, *Rdr4*, *Rdr5*, and *Rdr6*, have been identified and genetically mapped (Yasmin, 2011). Recent studies suggest that these *Rdr* genes are not functionally redundant but instead operate at distinct stages of infection or recognize different effectors, forming a hierarchical resistance network (Terefe-Ayana et al., 2011). Notably, expression of *muRdr1A* remains stable across different host genetic backgrounds. This observation suggests that the corresponding pathogen avirulence (Avr) factor is ubiquitous and relatively conserved within pathogen populations. Therefore, this Avr factor likely plays a central role in the interaction between *Rosa* spp. and black spot disease (Menz et al., 2018). The *Rdr4* gene confers resistance to multiple *D. rosae* races (2, 3, 8, 9, 10, 11, and 13) and exhibits predominantly dominant inheritance, enabling effective resistance in heterozygous plants (Zurn et al., 2018). By contrast, *Rdr3* confers strong resistance mainly to race 8, with only limited protection against other races, including races 3 and 9. Consequently, breeding programs often require pyramiding additional resistance genes, such as *Rdr4*, to achieve broader and more durable disease resistance (Zurn et al., 2020).

*CPR5* is recognized as a key regulatory factor that governs the balance between plant growth and defense responses (Meng et al., 2017). It modulates energy allocation between growth and defense mechanisms, enabling plants to dynamically adjust developmental and immune responses under stress conditions, particularly during pathogen attack. Such regulatory mechanisms are essential for plant survival and reproductive success. Under normal growth conditions, *RcCPR5* suppresses premature immune activation by forming homo- and heterodimers and interacting with *RcSIM*/*RcSMR* to assemble regulatory complexes, thereby prioritizing growth and developmental processes. Upon pathogen invasion, this inhibitory state is rapidly relieved, redirecting resources toward defense activation and enhancing resistance to infection. Downregulation of *RcCPR5* in *R. hybrida* confers resistance to powdery mildew and exhibits broad-spectrum resistance characteristics. These findings provide a promising molecular target for resistance breeding programs (Li et al., 2023a). *RcCPR5* appears to confer disease resistance through a mechanism that is highly conserved with that of CPR5 in *A. thaliana*. Both maintain immune homeostasis by forming complexes with SIM/SMR-like cell-cycle inhibitors, and, moreover, upon pathogen infection, they release SIM/SMR through complex dissociation, thereby activating ETI-related defense responses (Wang et al., 2014a). In contrast, *RcCPR5* has two alternatively spliced isoforms that can form both homodimers and heterodimers for jointly regulating immune activation. Thus, an evolutionary expansion of CPR5-mediated immune regulation in woody ornamental plants can be revealed.

*MLO* (Mildew Locus O) genes encode a class of membrane proteins with seven transmembrane domains and are widely regarded as susceptibility factors required for plant infection by powdery mildew pathogens (Appiano et al., 2015; Devoto et al., 2003; Kusch, Pesch & Panstruga, 2016). In barley, *A. thaliana*, and other plants, the *MLO*-mediated susceptibility mechanism is highly conserved in both monocot and dicot species; MLO proteins mainly promote pathogen infection by suppressing papilla formation, regulating ROS accumulation, affecting vesicle trafficking, and modulating Ca^2+^ signal transduction (Bhat et al., 2005; Consonni et al., 2006; Kim et al., 2002; Piffanelli et al., 2002). It is noteworthy that the copy number of *MLO* genes, the degree of functional redundancy, and the fitness costs associated with their inactivation vary considerably among species. In general, loss of *MLO* function confers durable and broad-spectrum mlo-based resistance (Acevedo-Garcia et al., 2017; Kusch & Panstruga, 2017).

In *R. hybrida*, Fang et al. (2021), Qiu et al. (2015) identified 19 members of the *RhMLO* gene family and demonstrated through functional analyses that *RhMLO1* and *RhMLO2* belong to the susceptibility-associated Clade V. Silencing of *RhMLO1* or *RhMLO2* significantly reduced the infectivity of the powdery mildew pathogen and enhanced disease resistance, indicating that these genes play important roles in mediating susceptibility to powdery mildew in *R. hybrida*.

In contrast with model plants and cereal crops, the functional characterization of the *MLO* family in rose remains in its infancy. Although several *RhMLO* candidates have been implicated in powdery mildew susceptibility, the molecular mechanisms of their functions remain unclear. Subsequent research integrating functional genomics and genome-editing technologies will be required for elucidating *RhMLO*-mediated susceptibility pathways and exploiting their potential in breeding broad-spectrum disease-resistant rose cultivars.

The plant cell wall not only provides structural support but also functions as a primary defense barrier during invasion by pathogenic fungi. The expansin (EXP) family, a class of proteins that regulates cell wall loosening and remodeling, plays important roles in plant growth, development, and disease resistance (Zhang et al., 2025b). A total of 31 *RcEXP* genes have been identified in the *R. chinensis* genome and are unevenly distributed across seven chromosomes.

*RcEXPA8*, a member of the expansin (EXP) family in *R. chinensis* negatively regulates host resistance during black spot infection. Silencing *RcEXPA8* significantly enhanced resistance to black spot disease in roses (Zheng et al., 2024c). Similar observations have been reported in *A. thaliana*, where RNA interference-mediated suppression of *AtEXLA2* markedly increased plant resistance to the necrotrophic fungi *B. cinerea* and *A. brassicicola*. Mechanistically, EXLA2-mediated susceptibility is associated with the COI1-dependent JA signaling pathway, and EXLA2 has been shown to play a key regulatory role in cyclopentenone oxylipid-mediated defense responses against *B. cinerea*. These findings suggest that expansins not only influence the physical properties of the cell wall but also modulate downstream immune signaling networks (Abuqamar et al., 2013). In *O. sativa*, auxin-inducible *OsEXPB3*, *OsEXPB4*, and *OsEXPB7* facilitate pathogen infection by promoting cell wall loosening. It is noteworthy that GH3-8-mediated auxin inactivation represses EXP expression while strengthening basal immunity (Ding et al., 2008). There has been limited direct evidence linking *TaEXPB23* to disease resistance in wheat. However, its notable induction by methyl jasmonate (MeJA) and localization to the cell wall suggest a potential involvement in defense-associated cell wall remodeling (Han et al., 2012). The above findings support a conserved model in which expansins serve as key regulators connecting cell wall dynamics with immune responses. While controlled cell wall remodeling is essential for growth and stress adaptation, excessive expansin activity may weaken structural barriers and suppress defense-associated signaling, thereby enhancing susceptibility to pathogen invasion. The above research lays a vital theoretical foundation for investigating the disease-resistance functions of the EXP gene family in *R. chinensis*.

The SABATH gene family encodes a class of S-adenosyl-L-methionine-dependent methyltransferases that catalyze the methylation of signaling molecules such as SA, benzoic acid (BA), and JA, thereby playing important regulatory roles in plant defense responses. This gene family exhibits both structural conservation and functional diversification and has undergone tandem duplication during evolution. As indicated by existing research, that *AtBSMT1* in *A. thaliana* and other SABATH family members in diverse plant species can catalyze the conversion of SA to methyl salicylate (MeSA) (Chen et al., 2003; Ross et al., 1999). MeSA, a key mobile signal in SAR, is capable of transmiting immune information between local infection sites and distal tissues, thereby promoting defense gene expression and the establishment of whole-plant resistance (Park et al., 2007). Meanwhile, some SABATH family methyltransferases also function as jasmonic acid carboxyl methyltransferases (JMTs), converting JA to MeJA and thereby regulating defense responses induced by necrotrophic pathogens (Seo et al., 2001).

Compared with model plants and cereal crops, research on the disease-resistance functions of the SABATH family in rose has lagged behind. A recent genome-wide study identified 47 *RcSABATH* members in *R. chinensis* and showed that *RcSABATH20* exhibited an expression pattern closely associated with resistance to rose black spot disease. *RcSABATH20* was expressed at significantly higher levels in the resistant line than in the susceptible line, and exogenous SA treatment induced its expression. Nevertheless, the SA biosynthesis inhibitor AIP suppressed its transcript accumulation. As revealed by the above-mentioned results, *RcSABATH20* may participate in SA-mediated defense regulation (Zheng et al., 2024a). In *A. thaliana*, the SABATH gene family plays a central role in disease resistance by regulating SA-MeSA-mediated SAR and maintaining the balance between SA and JA signaling. Aligning with the above view, *AtBSMT1* exhibits temporal and spatial regulation and is induced by disease-related stimuli and JA signaling. However, it is noteworthy that overexpression of salicylic acidcarboxyl methyltransferase (SAMT) reduces SA-mediated pathogen resistance in *A. thaliana* (Koo et al., 2007; Sendon et al., 2012). As indicated by the functional characteristics of the SABATH family in *A. thaliana*, *RcSABATH20* may not merely a gene responsive to SA signaling, but rather a regulator involved in SA metabolism, MeSA biosynthesis, as well as SAR establishment. Notably, recent studies on rose black spot disease have confirmed that SA signaling is an important defense pathway against *M. rosae*, and that multiple disease-resistance-related genes participate in immune regulation by modulating SA accumulation and SA-JA crosstalk. Therefore, *RcSABATH20* can be of critical significance in the SA signaling amplification network, influencing the establishment of local immunity and systemic resistance by regulating the dynamic balance between SA and its derivatives. It is imperative to carry out subsequent research combining enzymatic characterization, VIGS-mediated gene silencing, overexpression analysis, and metabolomic profiling to determine the substrate specificity of *RcSABATH20* and its impact on MeSA accumulation. Accordingly, the molecular mechanism of the “SABATH-SA/MeSA-SAR” defense module in rose can be elucidated, and new candidate genes can be provided for molecular breeding of black spot disease resistance.

## Main molecular approaches for disease-resistance breeding in roses

### Genome-wide characterization of *Rosa* species

The continuous advancement of high-throughput sequencing platforms, including Illumina short-read sequencing, PacBio Single-Molecule Real-Time (SMRT) sequencing, and Oxford Nanopore long-read sequencing, in conjunction with optimized assembly algorithms, has substantially improved the quality and completeness of the *R. chinensis* genome assembly (Giani et al., 2020). The *R. chinensis* genome exhibits high heterozygosity and polyploidy, alongside a complex hybrid origin, which poses considerable challenges for genomic research (Wylie, 1954; Zhang et al., 2023). While most wild *R. chinensis* individuals are diploid, modern cultivated varieties are predominantly tetraploid, with their genomes originating from a mix of 8 to 20 ancient wild species and cultivars (Wylie, 1954). The cultivar ‘Old Blush’, which introduced continuous flowering into the previously once-flowering European rose, is recognized as a key ancestral contributor to modern *R. chinensis* (Raymond et al., 2018).

Hibrand Saint-Oyant et al. (2018) sequenced a doubled haploid line derived from the diploid cultivar ‘Old Blush’ and integrated long-read and short-read sequencing data to construct a high-quality genome assembly anchored to seven pseudochromosomes. Their study clarified the genomic architecture governing core ornamental traits of rose, including flower color (anthocyanin biosynthesis), fragrance (terpenoid and phenylpropanoid metabolism), petal morphology (APETALA2/TOE family genes), and flowering time (*e.g*., *TFL1* and *AP1*). Zhang et al. (2024c) performed high-resolution haplotype assembly and population resequencing on the tetraploid *R. chinensis* cultivar ‘Samantha’, and the results revealed that ‘Old Blush’ and *Rosa odorata* (and its derivatives) serve as the primary genomic contributors to modern *R. chinensis*. Although wild species including *Rosa multiflora* (*R. multiflora*) and *Rosa rugosa* (*R. rugosa*) contribute a relatively small proportion of the genome overall, they supply abundant functional variations in genomic regions associated with stress resistance (Zhang et al., 2024c). Zhang et al. (2024a) generated a high-precision haplotype-resolved genome assembly to comprehensively characterize the structure and variation of the diploid *R. chinensis* genome, and integrated multi-tissue transcriptomic data to profile allele-specific gene expression under pathogen infection. This research lays a solid foundation for identifying alleles directly responsible for disease resistance. The completion of the *R. chinensis* whole-genome sequence has provided abundant genomic resources for deciphering the genetic mechanisms underlying key ornamental traits, including flower color, fragrance, and recurrent flowering. Furthermore, it supports the identification of functional genes and facilitates molecular breeding, thereby accelerating the genetic improvement of *R. chinensis* cultivars (Hibrand Saint-Oyant et al., 2018). Collectively, these research advances deepen our understanding of the evolutionary dynamics of rose disease resistance during domestication and breeding, and provide valuable candidate genes for the precise improvement of disease resistance *via* marker-assisted selection and gene-editing strategies (Smulders et al., 2019).

Moreover, the application of next-generation sequencing (NGS) technologies has profoundly advanced research on disease resistance in roses. The availability of high-quality reference genomes enables comparative genomic analyses to identify disease resistance genes and structural variations among cultivars. Wang et al. (2025b) integrated Illumina, PacBio, and Hi-C sequencing technologies to generate a high-quality chromosome-level assembly of *Rosa laevigata* (“Dog Rose”), providing a valuable resource for comparative genomics and genome-assisted disease resistance breeding. Likewise, Liu et al. (2024) combined PacBio HiFi, Oxford Nanopore ultra-long reads, and Hi-C data to develop a telomere-to-telomere continuous assembly of *R. rugosa* (“Tea Rose”). Their results indicated that recent tandem repeat expansions contribute significantly to stress tolerance, including disease resistance. These high-quality assemblies enable genome-wide quantitative trait locus (QTL) mapping and structural variation analyses for disease resistance traits. Cheng et al. (2025b) reported the first tetraploid *R. chinensis* genome assembly comprising 14 pseudochromosomes, providing essential resources for constructing tetraploid genetic maps and identifying disease resistance loci. In combination with analytical tools such as FlexQTL and polymapR, multi-parent population analyses have facilitated the identification of major QTLs associated with resistance traits, including leaf spot and black spot diseases (Rawandoozi et al., 2022). The above-mentioned advances underpin the development of SNP markers associated with disease resistance and facilitate genome-editing applications, thereby enabling the precise improvement of rose disease resistance through marker-assisted selection and gene-editing strategies.

### The application of CRISPR/CAS9

In recent years, precise gene-editing technologies, including CRISPR/Cas9 based on sgRNA-Cas complexes, have been maturing. Researchers have used the sequence-specific recognition and double-strand break (DSB) repair mechanisms of CRISPR/Cas9 to efficiently introduce insertion/deletion (InDel) mutations into crop genomes. This approach enables highly efficient targeted gene knockout. To improve rice resistance to bacterial blight caused by *Xanthomonas oryzae* pv. oryzae, Zeng et al. (2020) employed CRISPR/Cas9 to disrupt the susceptibility gene *OsSWEET14*. The edited lines exhibited broad-spectrum resistance to both Asian and African pathogen strains while maintaining yield performance, demonstrating great potential for molecular breeding applications. Dreiseitl (2024) precisely targeted the conserved regions of the Mlo gene family in gramineous and rosaceous crops *via* a multiplex sgRNA design combined with high-fidelity Cas9 variants and efficiently identified homozygous knockout lines using fluorescence-based reporter systems. In parallel, tandem TALEN technology has been applied to simultaneously target homologous loci, thereby overcoming functional redundancy among gene family members. The edited lines show normal growth and development as well as stable, broad-spectrum resistance to multiple powdery mildew pathogens. Wang et al. (2014b) demonstrated that TALEN-induced mutations in all three *Triticum aestivum MLO* homoeologs within a single plant confer heritable broad-spectrum resistance to powdery mildew. Multi-year field evaluations have confirmed the durability and effectiveness of this resistance, underscoring the strategic value of CRISPR/Cas9 and TALEN technologies in developing crop varieties with durable disease resistance and improved regulatory acceptability.

With continuous advances in gene-editing technologies, CRISPR/Cas9 holds considerable promise for applications in *Rosa* species, particularly for the improvement of fragrance traits and disease resistance. Wang et al. (2023) utilized the Arabidopsis U6-29 promoter to drive single guide RNA (sgRNA) expression, which significantly optimized the editing system. They also established a workflow for screening highly efficient sgRNAs and successfully constructed an efficient CRISPR/Cas9 genome-editing platform for *R.hybrida*. This optimized platform covers multiple procedural steps, including explant selection, hormone combination optimization, and screening pressure setting. Using this system, researchers successfully targeted and edited *RhEIN2*, a key gene involved in the ET signaling pathway; the resulting rose plants exhibited an ET-insensitive phenotype, which strongly verifies the efficacy of the established platform. This study is considered a key milestone in the field of rose genome editing. Notably, the application of CRISPR/Cas9 in *Roses* has lagged far behind that in staple crops such as rice and tomato, primarily because most commercial rose cultivars are highly heterozygous polyploids and lack efficient, genotype-independent regeneration and transformation systems. Previous studies have reported that the highest recorded transformation and regeneration efficiency in rose is only approximately 12%, and in most cases, this value is considerably lower (Smulders et al., 2019). In diploid organisms, the complete loss of gene function can usually be achieved by simultaneously knocking out both alleles, thereby generating readily observable phenotypes (Vaia et al., 2022). However, in tetraploid roses, all four copies of a target gene theoretically require simultaneous editing (Schaart, van de Wiel & Smulders, 2021). If the CRISPR/Cas9 system edits only one or two of these gene copies, the remaining wild-type copies can still synthesize functional proteins, resulting in phenotypes indistinguishable from or only slightly different from those of wild-type plants (Vaia et al., 2022). In addition, *Rosa* species possess highly complex genetic backgrounds: modern rose cultivars originate from interspecific hybridization of multiple wild species, and successive rounds of crossing have induced extensive genetic introgression and recombination, which further fragment breeding target loci. Moreover, *in vitro* rose regeneration is highly genotype-dependent. The few successful cases reported to date are restricted to a limited number of cultivars, and regeneration and transformation protocols optimized for one laboratory-adapted cultivar are highly likely to be ineffective when applied to other commercially valuable cultivars (Nelson et al., 2025). Beyond the inherent biological limitations of *Rosa* species themselves, researchers utilizing the CRISPR/Cas9 system in rose research also face several universal technical constraints of CRISPR/Cas9 technology, which are further exacerbated by the complex genetic background of roses. The large, repeat-rich polyploid rose genome enables Cas9 nuclease to cleave non-target loci with sequences homologous to the intended target sites, thus inducing unintended mutations (Vaghari-Tabari et al., 2022). This poses greater challenges for screening target sites with high specificity and correspondingly elevates the risk of off-target effects. Furthermore, the conventional SpCas9 nuclease requires an NGG protospacer adjacent motif (PAM) for cleavage, which restricts the editable range of the CRISPR/Cas9 system (Vaghari-Tabari et al., 2022; Zang et al., 2026). When precise editing of a specific SNP site is needed, or when a common target need to be identified among highly heterozygous alleles, PAM dependency may hinder the design of effective sgRNA.

### Quantitative trait loci (QTL)-based marker-assisted selection (MAS) platform

Quantitative trait loci (QTLs) are genomic regions that exhibit statistically significant associations with variations in complex traits, including yield, disease resistance, and quality. These loci are typically identified *via* high-density molecular marker genotyping combined with interval mapping or association analyses in segregating populations (*e.g*., F₂, RIL, DH) (Platten, Cobb & Zantua, 2019). Marker-assisted selection (MAS) utilizes molecular markers, such as SSRs, SNPs, and InDels, that are tightly linked to QTLs to screen breeding populations at any developmental stage. This strategy enables the rapid identification and selection of individuals carrying favorable alleles, thereby accelerating breeding cycles and reducing the costs and labor associated with extensive field-based phenotyping (Koebner, 2003). The integration of QTL mapping and MAS improves breeding efficiency while offering a robust framework for precisely improving complex traits (Raj & Nadarajah, 2022).

In *R. chinensis*, Lau et al. (2023) constructed two tetraploid linkage maps associated with resistance to black spot and leaf spot diseases. QTLs conferring resistance to black spot were identified on linkage groups (LGs) 1, 3, 5, and 6 in one population and on LGs 1, 2, 4, and 5 in another population. A major QTL on LG 5 co-localized with the black spot resistance gene *Rdr4*, suggesting that this locus likely corresponds to the genetic signal of *Rdr4*. QTLs associated with leaf spot resistance were detected on LGs 1 and 5 in one population and on LGs 1, 4, and 5 in another population. Notably, QTLs for both black spot resistance and leaf spot susceptibility were mapped to LG 5. Accordingly, the potential presence of either a single pleiotropic gene or two tightly linked genes that exert opposing effects on these traits could be indicated (Lau et al., 2023; Zurn et al., 2020). Rose rosette disease is the most destructive viral disease affecting *R. chinensis* in the United States, and no definitive or effective treatment is currently available (Dobhal et al., 2016). Based on QTL mapping analyses in both diploid and tetraploid *R. chinensis*, Hochhaus et al. (2023) localized resistance-associated QTL confidence intervals to LG 5 and LG 6. Genome-wide annotation and functional prediction analyses have preliminarily identified candidate genes potentially involved in disease resistance. The above-mentioned findings provide direct targets (gene editing, overexpression, and gene silencing) for subsequent functional validation while facilitating the development of marker-assisted selection tools.

## Discussion

*Roses* is threatened by several economically important fungal diseases (*e.g*., gray mold, black spot, powdery mildew, and downy mildew). These diseases can severely reduce ornamental quality, flowering performance, and commercial value worldwide (Debener & Byrne, 2014; Mekapogu et al., 2021). The above-mentioned pathogens differ in their infection strategies, host specificity, and environmental requirements. Notably, they collectively exploit host physiological processes to establish colonization and promote disease development (Domínguez-Serrano et al., 2016; Veloso & van Kan, 2018). As indicated by increasing evidence, phytohormone-mediated responses, particularly those involving SA, JA, ET, CK, and ABA, are of critical significance in determining disease outcomes during interactions between roses and fungal pathogens (Cao et al., 2019; Liu et al., 2022; Yang et al., 2022). However, current knowledge remains heavily centered on a limited number of classical phytohormones and a few model pathosystems, particularly *B. cinerea*, *P. pannosa*, and *M. rosae*. By contrast, the functions of several emerging defense-related hormones, encompassing brassinosteroids (BRs), gibberellins (GAs), auxin, strigolactones (SLs), and peptide hormones, remain largely unexplored in *Rosa* species, despite growing evidence from model plants demonstrating that these signaling pathways substantially regulate plant immunity through extensive hormonal crosstalk and growth-defense trade-offs. Moreover, fungal pathogens exhibit high transmissibility, and breeding for disease resistance in *R. chinensis* must be integrated with tolerance to abiotic stresses such as drought, salinity, and heat (Bheemanahalli et al., 2021).

In rose, disease resistance manifests as a layered molecular network in which WRKY, MYB, bHLH, and bZIP factors, together with Rdr/NLR genes and hormone- or cell wall-related regulators, coordinate SA-JA crosstalk, ROS homeostasis, and structural defense (Ding et al., 2023; Hu et al., 2021; Ren et al., 2020; Zheng et al., 2024b). However, most current rose studies still rely on transcriptomic changes or single-gene silencing in a single cultivar; thus, these studies demonstrate “association” rather than “causation,” and their conclusions are mostly limited to gray mold instead of the broader spectrum of economically important pathogens. For example, the Rdr4 locus has been shown to confer resistance to multiple black spot races, yet the causal gene has not been fully characterized, and its durability requires pyramiding with other resistance loci (Yasmin, 2011). Likewise, Fang et al. (2021) identified *RhMLO1/2* as powdery mildew susceptibility genes, while the functions of *RcEXPA8* and *RcSABATH20* are only supported by gene expression and gene silencing evidence. Their direct biochemical mechanisms, target interactions, and functional performance across diverse germplasm remain unresolved. Accordingly, future research requires more efforts, including advancing beyond candidate confirmation to clone causal genes, defining downstream targets and protein networks, and testing multiple rose genotypes and pathogen races under field-relevant conditions, with the aim to identify durable, broadly applicable regulatory factors for breeding.

Whole-genome resequencing enables the identification of genotypes or breeding lines harboring favorable haplotypes or alleles, particularly from underutilized genetic resources including wild relatives, local landraces, and ex situ germplasm collections. These beneficial genetic variants serve as valuable sources of durable disease resistance in breeding programs. Cultivated *R. chinensis* are typically tetraploid (2n = 4x) and frequently exhibit complex polyploid genomic characteristics, including tetrasomic inheritance and allele dosage variation. As a result, sequence divergence among alleles and variations in protospacer adjacent motif (PAM) sequences may hinder a single CRISPR/Cas9 single-guide RNA (sgRNA) from efficiently targeting all homoeologous gene copies. Such incomplete targeting can lead to partial phenotypic modification, given that functional redundancy of unedited alleles may mask the intended trait alteration (Assa et al., 2024). Furthermore, breeding strategies and functional validation that rely solely on linkage maps or analytical models established based on diploid genetic frameworks may overlook or underestimate critical polyploid-specific factors. These factors encompass allelic dosage effects, the contributions of multiple homoeoallelic loci, and recombination between homoeologous chromosomes. Therefore, to improve the accuracy and efficacy of molecular breeding and genome editing in polyploid species, multiple sgRNAs, or nucleases with expanded targeting scope, should be designed to achieve complete targeting of all allelic copies. Meanwhile, high-density linkage maps integrated with haplotype phasing should be constructed specifically for tetraploid populations. In addition, polyploid-adapted genotyping and population genetic approaches, including allele dosage estimation, high-coverage or long-read sequencing for haplotype phasing, and multi-allelic QTL and linkage analysis, should be implemented to accurately resolve genotype-phenotype associations and minimize erroneous interpretations (Wu, Liang & Byrne, 2019).

Moving forward, the integration of conventional breeding and molecular approaches will be essential for developing *R. chinensis* cultivars with robust, broad-spectrum disease resistance. Abundant genetic resources will support future genomic research and advance the understanding of genetic diversity. Such advances will improve the efficiency and precision of global *R. chinensis* breeding programs, facilitate low-maintenance cultivation, optimize urban landscape aesthetics, and ultimately reduce management costs.

## Additional Information and Declarations

### Data Availability

The following information was supplied regarding data availability:

This is a Literature Review.
